# NODDI-DTI: Estimating Neurite Orientation and Dispersion Parameters from a Diffusion Tensor in Healthy White Matter

**DOI:** 10.3389/fnins.2017.00720

**Published:** 2017-12-20

**Authors:** Luke J. Edwards, Kerrin J. Pine, Isabel Ellerbrock, Nikolaus Weiskopf, Siawoosh Mohammadi

**Affiliations:** ^1^Department of Neurophysics, Max Planck Institute for Human Cognitive and Brain Sciences, Leipzig, Germany; ^2^Wellcome Trust Centre for Neuroimaging, UCL Institute of Neurology, University College London, London, United Kingdom; ^3^Institute of Systems Neuroscience, University Medical Center Hamburg-Eppendorf, Hamburg, Germany

**Keywords:** diffusion MRI, NODDI, DTI, axonal density, orientation dispersion

## Abstract

The NODDI-DTI signal model is a modification of the NODDI signal model that formally allows interpretation of standard single-shell DTI data in terms of biophysical parameters in healthy human white matter (WM). The NODDI-DTI signal model contains no CSF compartment, restricting application to voxels without CSF partial-volume contamination. This modification allowed derivation of analytical relations between parameters representing axon density and dispersion, and DTI invariants (MD and FA) from the NODDI-DTI signal model. These relations formally allow extraction of biophysical parameters from DTI data. NODDI-DTI parameters were estimated by applying the proposed analytical relations to DTI parameters estimated from the first shell of data, and compared to parameters estimated by fitting the NODDI-DTI model to both shells of data (reference dataset) in the WM of 14 *in vivo* diffusion datasets recorded with two different protocols, and in simulated data. The first two datasets were also fit to the NODDI-DTI model using only the first shell (as for DTI) of data. NODDI-DTI parameters estimated from DTI, and NODDI-DTI parameters estimated by fitting the model to the first shell of data gave similar errors compared to two-shell NODDI-DTI estimates. The simulations showed the NODDI-DTI method to be more noise-robust than the two-shell fitting procedure. The NODDI-DTI method gave unphysical parameter estimates in a small percentage of voxels, reflecting voxelwise DTI estimation error or NODDI-DTI model invalidity. In the course of evaluating the NODDI-DTI model, it was found that diffusional kurtosis strongly biased DTI-based MD values, and so, making assumptions based on healthy WM, a novel heuristic correction requiring only DTI data was derived and used to mitigate this bias. Since validations were only performed on healthy WM, application to grey matter or pathological WM would require further validation. Our results demonstrate NODDI-DTI to be a promising model and technique to interpret restricted datasets acquired for DTI analysis in healthy white matter with greater biophysical specificity, though its limitations must be borne in mind.

## 1. Introduction

The white matter (WM) of the human brain consists of dense bundles of neuronal axons connecting the brain's functional areas. Neural circuits thus formed allow these areas to work together as a coherent entity. Changes in WM impact these neural circuits, and are thus the subject of studies investigating pathology (Acosta-Cabronero et al., 2010; Meinzer et al., 2010; Freund et al., 2013b), and cognition and learning (Scholz et al., 2009; Zatorre et al., 2012).

Diffusion tensor imaging (DTI; Basser et al., 1994; Jones, 2014) is, at present, the most commonly used method to observe WM changes *in-vivo* (Scholz et al., 2009; Fields, 2010; Zatorre et al., 2012). This is because DTI is simply implemented and time efficient while allowing robust estimation of complementary parameters [e.g., “fractional anisotropy” (FA) and “mean diffusivity” (MD), Pierpaoli et al., 1996] sensitive to microstructural WM changes (Beaulieu, 2014), even in clinical contexts (see e.g., Meinzer et al., 2010; Freund et al., 2013a). Despite its microstructural sensitivity, the model underlying DTI (gaussian anisotropic diffusion; Jones, 2014) is unspecific to biological changes. Numerous studies show MD and FA change in white matter [e.g., due to learning a new skill (Scholz et al., 2009) or the pathology of Alzheimer's disease (Acosta-Cabronero et al., 2010)], but cannot, in the absence of further information, distinguish e.g., changes in axon density from changes in axon arrangement.

In order to estimate parameters of direct neurobiological relevance from diffusion MRI, we need biophysical models (Beaulieu, 2014; De Santis et al., 2014; Seunarine and Alexander, 2014). The majority of biophysical models (including the model introduced below) are “multicompartment” diffusion models. Such models assume voxelwise diffusion contrast arises from linear combination of diffusion signals from distinguishable water compartments. Numerous multicompartment diffusion models have been proposed (see e.g., Stanisz et al., 1997; Jespersen et al., 2007, 2012; Fieremans et al., 2011; Panagiotaki et al., 2012; Sotiropoulos et al., 2012; Zhang et al., 2012; Jelescu et al., 2015; Kaden et al., 2016; Tariq et al., 2016), but the complexity and lack of robustness of most of these models hinder their routine use in neuroscientific and clinical studies.

The NODDI (neurite orientation dispersion and density imaging) model (Zhang et al., 2012) is a multicompartment model allowing robust and time-efficient estimation of maps of parameters representing neurite (in WM: axon) density and dispersion, and represents a trade-off between complexity, robustness, and acquisition-time duration. Robustness is achieved by fixing the values of several model parameters from earlier models (Zhang et al., 2011; Jespersen et al., 2012), reducing the number of fitted parameters. As a result, the amount of data required to invert the model is reduced, giving acquisition-time durations approaching those available in clinical settings (Zhang et al., 2012). NODDI is thus gaining popularity in diffusion application studies (Owen et al., 2014; Chang et al., 2015; Grussu et al., 2015; Jelescu et al., 2015; Wen et al., 2015; Tariq et al., 2016; Campbell et al., 2017), though the potential of fixed parameters to lead to bias in the fitted parameters has been a source of criticism (Jelescu et al., 2015, 2016; Kaden et al., 2016; Novikov et al., 2016).

Herein, we investigate a modification of the NODDI model in which the cerebrospinal fluid (CSF) compartment is not included, and derive explicit relations between the remaining parameters of this model and MD and FA. We call this modified version of the NODDI model the “NODDI-DTI” model because these relations formally allow extraction of neurite orientation and dispersion parameters using MD and FA from DTI. The existence of relations between NODDI-DTI and DTI parameters explains previously observed correlations between NODDI parameters and MD and FA (Zhang et al., 2012; Kunz et al., 2014; Grussu et al., 2015; Deligianni et al., 2016; Mayer et al., 2017). Practical application of the relations requires correction of the estimated DTI parameters for bias due to diffusional kurtosis, and so we derive and use a novel heuristic correction using only the DTI parameters to correct for this bias. We then examine the accuracy and limitations of estimating NODDI-DTI parameters from DTI parameters in WM.

## 2. Materials and methods

### 2.1. NODDI-DTI relations

The NODDI signal model supposes three compartments: intraneurite water, extraneurite water, and free water (Zhang et al., 2012). The biophysical parameters fitted in the model are neurite density (volume fraction of the intraneurite compartment), ν; a measure of neurite dispersion, κ; a vector giving the main neurite orientation; and a volume fraction accounting for partial-volume effects with free water (nominally CSF; Vos et al., 2011; Metzler-Baddeley et al., 2012; Zhang et al., 2012). An important fixed parameter is the intrinsic diffusivity of the intraneurite compartment, *d* = 1.7 × 10^−3^ mm^2^ s^−1^ (Zhang et al., 2012). The primary neurite orientation (Zhang et al., 2012) is formally equivalent to the principal eigenvector of the diffusion tensor (DT; see Appendix A), as Daducci et al. (2015) previously observed empirically.

The NODDI-DTI model is a reduced form of the NODDI model with no CSF volume fraction; this model has been previously observed to give reasonable estimates of ν and κ from single-shell data (Magnollay et al., 2014). For ease of computation in the following we use τ instead of κ as our measure of dispersion, where (Jespersen et al., 2012; Zhang et al., 2012; Jelescu et al., 2015)

(1) $$\begin{array}{r} \tau =\frac{1}{\sqrt{\pi \kappa }\exp\left(-\kappa \right)\text{erfi}\left(\sqrt{\kappa }\right)}-\frac{1}{2\kappa },\tau \in \left[1/3,1\right], \end{array}$$

and erfi is the imaginary error function. The parameter τ ranges from 1/3 (isotropically distributed neurites) to 1 (perfectly aligned neurites)—increasing τ corresponds to increasing neurite alignment—and is the average of cos^2^(ψ) over the neurite distribution, where ψ is the angle between a given neurite and the main neurite orientation (Jelescu et al., 2015).

By expanding the NODDI-DTI signal model in moments, one can derive a corresponding DT (Jespersen et al., 2012). As shown in Appendix A, appropriate combination of the eigenvalues of this DT allows expression of ν and τ in terms of MD and FA of this DT:

(2) $$\begin{array}{rcl} \nu & = & 1-\sqrt{\frac{1}{2}\left(\frac{3\text{MD}}{d}-1\right)}, \end{array}$$

(3) $$\tau =\frac{1}{3}\left(1+\frac{4}{\left|d-\text{MD}\right|}\frac{\text{MD}\cdot \text{FA}}{\sqrt{3-2{\text{FA}}^{2}}}\right).$$

We note that Equation (2) has been independently derived by Lampinen et al. (2017).

Equations (2) and (3) demonstrate a one-to-one mapping from (MD, FA) to (ν, τ), implying that, formally, NODDI-DTI parameters can be extracted from DTI data. We can predict domains within which MD and FA should lie if the NODDI-DTI model provides a valid representation: substituting ν∈[0, 1] and τ∈[1/3, 1] into Equations (2) and (3) gives the domains

(4) $$\text{MD}\in [d/3,d],\text{ FA}\in \left[0,\sqrt{\frac{3}{2}}\frac{\left|d-\text{MD}\right|}{\sqrt{2{\text{MD}}^{2}+{\left(d-\text{MD}\right)}^{2}}}\right].$$

Values of MD and FA lying outside of these bounds will give rise to unphysical ν and τ estimates, and could result either from errors in quantifying the DT (i.e., the first moment of the diffusion signal), or from the invalidity of the NODDI-DTI model as a representation in a given voxel.

### 2.2. Heuristic correction for diffusional kurtosis

The experimental diffusion signal contains contributions from moments higher than the diffusion tensor. These higher order moments bias MD estimates from typical (single-shell) DTI data (Veraart et al., 2011), meaning that the experimentally determined MD is not completely analogous to the theoretical MD in Equations (2) and (3). The bias can be strongly mitigated using data measured at more than two *b*-values (multi-shell data), but this extra measurement is usually not practical (Veraart et al., 2011). In order to mitigate this bias without requiring extra data, we define the heuristically corrected MD,

(5) $${\text{MD}}_{\text{h}}=\text{MD}+\frac{b}{6}\left(\sum_{i,j=1}^{3}\frac{1+2\delta_{ij}}{15}\lambda_{i}\lambda_{j}\right),$$

where λ*i* is the *i*th eigenvalue of the measured DT and δ_*ij*_ is the Kronecker delta. Equation (5), derived in Appendix B, pragmatically assumes that only the first higher moment, diffusional kurtosis (Jensen and Helpern, 2010), contributes; that the square of the apparent diffusion coefficient is uncorrelated with the apparent diffusional kurtosis; that the mean diffusional kurtosis can be taken to be unity [approximately true over much healthy human brain WM (Jensen and Helpern, 2010; Lätt et al., 2013; André et al., 2014; Mohammadi et al., 2015)]; and that the effect of diffusional kurtosis on each individual eigenvalue is negligible. Substituting Equation (5) into Equation (2) then gives the relation used in the following to estimate ν from experimentally determined DT invariants:

(6) $$\begin{array}{l} \nu =1-\sqrt{\frac{3{\text{MD}}_{\text{h}}}{2d}-\frac{1}{2}}\\ \;=\;1-\sqrt{\frac{3}{2d}\left(\text{MD}+\frac{b}{6}\left(\sum_{i,j=1}^{3}\frac{1+2\delta_{ij}}{15}\lambda_{i}\lambda_{j}\right)\right)-\frac{1}{2}}. \end{array}$$

The effect of failing to correct for diffusional kurtosis is much less pronounced for FA (Veraart et al., 2011), and preliminary experiments (data not shown) showed that applying diffusional kurtosis correction to only MD in Equation (3) resulted in a modest increase in the number of unphysical τ parameter estimates. This latter observation can be explained using Equation (4): whenever heuristic diffusional kurtosis correction leads to overestimation of MD, the upper bound for allowed FA values is artificially decreased, potentially leading to unphysical τ estimates. We therefore apply no correction to Equation (3).

### 2.3. Data collection and preprocessing

All data were collected by scanning healthy volunteers in a MAGNETOM Tim Trio 3 T MRI system (Siemens AG, Healthcare Sector, Erlangen, Germany). The investigation involving the first 2 subjects was carried out in accordance with the recommendations of “Quality assurance and optimization of magnetic resonance imaging sequences and processing for non-invasive neuroimaging in human subjects,” approved by the NRES Committee London - Queen Square. The investigation involving the group of 12 subjects was carried out in accordance with the recommendations of ethics agreement number PV5141, approved by the Ärztekammer Hamburg. All subjects gave written informed consent in accordance with the Declaration of Helsinki.

The data from subjects 1 and 2 were used to validate the heuristic diffusional kurtosis correction and provide preliminary validation of the stability of the NODDI-DTI model and method. The data from subjects 3–14, recorded with a different protocol, complemented the analyses of the data from subjects 1 and 2 by allowing insight into whether the behaviour of the NODDI-DTI method is stable over a wider number of subjects.

The first two datasets (subjects 1 and 2) were recorded using a 2D multiband spin-echo echo-planar imaging (EPI) sequence supplied by the Center for Magnetic Resonance Research, University of Minnesota (Moeller et al., 2010; Setsompop et al., 2012; Xu et al., 2013). Sequence parameters: field of view (FoV): 220 × 220 mm^2^, 81 slices, 1.7 mm isotropic resolution, echo time: TE = 112 ms, volume repetition time: TR = 4, 835 ms, partial Fourier factor: 6/8, multiband factor (Setsompop et al., 2012): 3, total 4 × 66 EPI images with 60 diffusion weighted images per shell using *b*-values of *b* = {1, 000;2, 500} s mm^−2^, and 6 non-diffusion weighted (*b* = 0) images per shell interleaved between the weighted acquisitions, 2 × phase encoding polarities (Anterior → Posterior/Posterior → Anterior).

The last 12 datasets (subjects 3–14) were recorded using a twice-refocused spin-echo EPI sequence also supplied by the Center for Magnetic Resonance Research, University of Minnesota (Reese et al., 2003). These datasets were previously used by Ellerbrock and Mohammadi (2018). Sequence parameters: FoV: 224 × 224 × 138 mm^3^, 86 slices, 1.6 mm isotropic resolution, TE = 122 ms, TR = 7, 100 ms, parallel imaging factor (Griswold et al., 2002): 2, partial Fourier factor: 7/8, multiband factor (Setsompop et al., 2012): 2, total 4 × 66 EPI images with 60 diffusion weighted images per shell using *b*-values of *b* = {1, 000;2, 000} s mm^−2^, and 6 interleaved *b* = 0 images per shell, 2 × phase encoding polarities (Anterior → Posterior/Posterior → Anterior).

For subjects 1 and 2, subject motion, eddy currents, and susceptibility distortions were corrected for using the ACID toolbox (http://www.diffusiontools.com/); for details see Mohammadi et al. (2010), Mohammadi et al. (2015), Ruthotto et al. (2012), and Ruthotto et al. (2013). The corrected data from the two phase encoding directions were then summed for use in subsequent analysis.

Subjects 3–14 were preprocessed by using the ACID toolbox to perform the following four step procedure:

1. For each shell, the first *b* = 0 image with reversed phase encoding was coregistered to the *b* = 0 image of the original phase encoding direction, and the resulting transformation was applied to all data with reversed phase encoding.
2. The data were then subjected to motion correction using a multi-target registration approach similar to that in Mohammadi et al. (2015). Eddy current distortion correction was not performed because the acquisition protocol employed parallel imaging and a twice-refocused spin echo scheme such that these distortions were negligible.
3. The data were corrected for susceptibility distortion artifacts by using the *b* = 0 images acquired with opposed phase encoding directions to estimate the fieldmap, and then using this fieldmap to unwarp all images (Ruthotto et al., 2012, 2013; Macdonald and Ruthotto, 2016).
4. As a final step, the mean of the corrected data from the two phase encoding directions was taken for use in subsequent analysis.

### 2.4. Parameter estimation and comparison

Parameters were estimated only in WM voxels determined to be largely unaffected by CSF or grey matter partial volume effects. This determination was made by thresholding at 50% probability a WM probability map obtained by segmenting the first *b* = 0 image of each respective preprocessed dataset in SPM12 (Wellcome Trust Centre for Neuroimaging, London, UK).

The ACID toolbox was used to estimate FA, MD, and the eigenvalues of the DT from the low *b*-value shell of each dataset, and in-house SPM scripts were then used to generate ν and τ using Equations (3) and (6), respectively, from these DT parameters; we refer to these as the “NODDI-DTI method” results. For subjects 1 and 2, the ACID toolbox was also used to simultaneously estimate the diffusion and kurtosis tensors (Mohammadi et al., 2015), giving silver standard mean diffusivity estimates (MD_DKI_) less biased by the effects of diffusional kurtosis (Veraart et al., 2011), allowing evaluation of the validity of Equation (5).

The effect of noise on NODDI-DTI parameter estimates was investigated using Matlab (R2013a, MathWorks, Natick, Mass., USA) simulations. Data *S*(ν, τ) were simulated with the NODDI toolbox v0.9 (http://www.nitrc.org/projects/noddi_toolbox/) using the diffusion protocol of subjects 1 and 2 for parameters typical of the corpus callosum (Jelescu et al., 2015): ν = 0.5, τ = cos^2^(18°) ≈ 0.9. The main fibre orientation was taken to be along (0, 0, 1)t, where ·t denotes the transpose operation. Rician noise (Henkelman, 1985; Gudbjartsson and Patz, 1995) was then added to the data such that the data became *S*′(ν, τ) = |*S*(ν, τ)+*l*+i*m*|, where *l* and *m* were each drawn from a Gaussian distribution of mean zero and standard deviation 1/[5, 20, 40], and i is the imaginary unit. Because the intensity of the simulated *b* = 0 signals was unity, this choice of standard deviation gave a respective signal to noise ratio (SNR) ≈ [5, 20, 40] for the *b* = 0 signals (Henkelman, 1985); these are typical SNR values for data used in diffusion imaging experiments (Jones and Basser, 2004).

Parameters were estimated from the noise-corrupted simulated data by linearly fitting a diffusion tensor to the log-transformed first shell of data and then applying Equations (3) and (6), and also by using the NODDI toolbox to fit the NODDI-DTI model to both shells of data [converting the fitted *kappa* to τ using Equation (1)]. In order to test whether the NODDI-DTI method ν estimate could be improved by taking into account the diffusional kurtosis bias explicitly, both shells of data were also fit to log(*S*′(ν, τ)) = −*b*MD+*b*^2^*C* by solving log(*S*′(ν, τ)) = (−*b, b*^2^)(MD, *C*)t using Matlab's backslash function; this gave an MD estimate less biased by higher order diffusion moments. Estimation of spherically averaged diffusion parameters in such a manner has been reported previously (Kaden et al., 2016; Novikov et al., 2016). This MD estimate was converted to an estimate of ν using Equation (2). The above procedure was repeated 1 × 10^3^ times for each SNR value, and the parameter estimations were also carried out for the noiseless original data. Histograms were then plotted of the parameter estimates from these repetitions in Matlab (R2017a) to allow analysis of the accuracy of the various methods in the presence and absence of noise. Histogram widths were those chosen by Matlab's histogram function for the SNR ≈ 5 NODDI-DTI method parameter estimates. Unphysical NODDI-DTI method parameter estimates are given as percentages of the total number of trials, and were not included when plotting the histograms.

For each subject, NODDI-DTI silver-standard results were obtained by fitting both shells of data using the NODDI toolbox with CSF volume fraction fixed at zero, followed by conversion of κ into τ using an in-house SPM script implementing Equation (1). We refer to these results as the “two-shell NODDI toolbox fitted” results, and they represent a silver-standard because two shells of data are sufficient to make inversion of multi-compartment signal models well-posed (Taquet et al., 2015). We exclude the CSF compartment in all cases in order to avoid the known overestimation of CSF volume fraction in WM found when using the NODDI model (Zhang et al., 2012; Cercignani and Bouyagoub, 2017).

In order to investigate the magnitude of the differences between NODDI-DTI method- and the NODDI toolbox fitted-results, for subjects 1 and 2 fits were also made using the NODDI toolbox of the subset of the diffusion data used for the DTI fitting. The designation “one-shell NODDI toolbox fitted” distinguishes these results from the NODDI-DTI method- and two-shell NODDI toolbox fitted-results. The NODDI toolbox has been shown previously to give reasonable results fitting single-shell data when the CSF compartment fraction is fixed at zero (Magnollay et al., 2014).

Parameter estimate comparisons were quantified using means and standard deviations of the differences, visualised using Bland–Altman plots (Bland and Altman, 1986). Estimates of ν and τ from Equations (3) and (6) which were unphysical (i.e., ν∉[0, 1], τ∉[1/3, 1]) were excluded from these statistical analyses. For subjects 1 and 2, Bland–Altman plots were generated for each dataset and parameter. Data from subjects 3–14 were combined in one Bland–Altman plot in order to investigate the stability of the differences over a larger group. The number of unphysical parameter estimates is given as a percentage of the total number of WM voxels for each subject.

Images of the mean difference between the NODDI-DTI method- and the NODDI toolbox fitted-results were also generated in order to provide a compact visual representation of the behaviour of the NODDI-DTI method in subjects 3–14. In order to facilitate warping the data to a standard group space, unphysical parameter estimates in the maps derived using the NODDI-DTI method were replaced with the average from the six closest voxels, excluding other unphysical parameter estimates and voxels outside each subject's WM mask. The replacement process was iterated until there was no change in the number of unphysical parameters; the remaining unphysical estimates (representing unconnected voxels in the WM mask) were arbitrarily replaced with 0 in ν maps and 1/3 in τ maps. Using the SPM12 normalisation tool, the first *b* = 0 image of each dataset was then used to generate transformations between the diffusion data and the ICBM European standard template at an isotropic resolution of 2 mm, which were subsequently applied to the NODDI-DTI method- and the NODDI toolbox fitted-maps to transform all maps into the standard group space. The mean difference over the whole group was then computed voxelwise for each parameter. Non-WM voxels were masked out for plotting by requiring that the WM probability for a given voxel be greater than 0.5 in the standard-space tissue probability mask provided by SPM12.

## 3. Results

**Figure 1:**
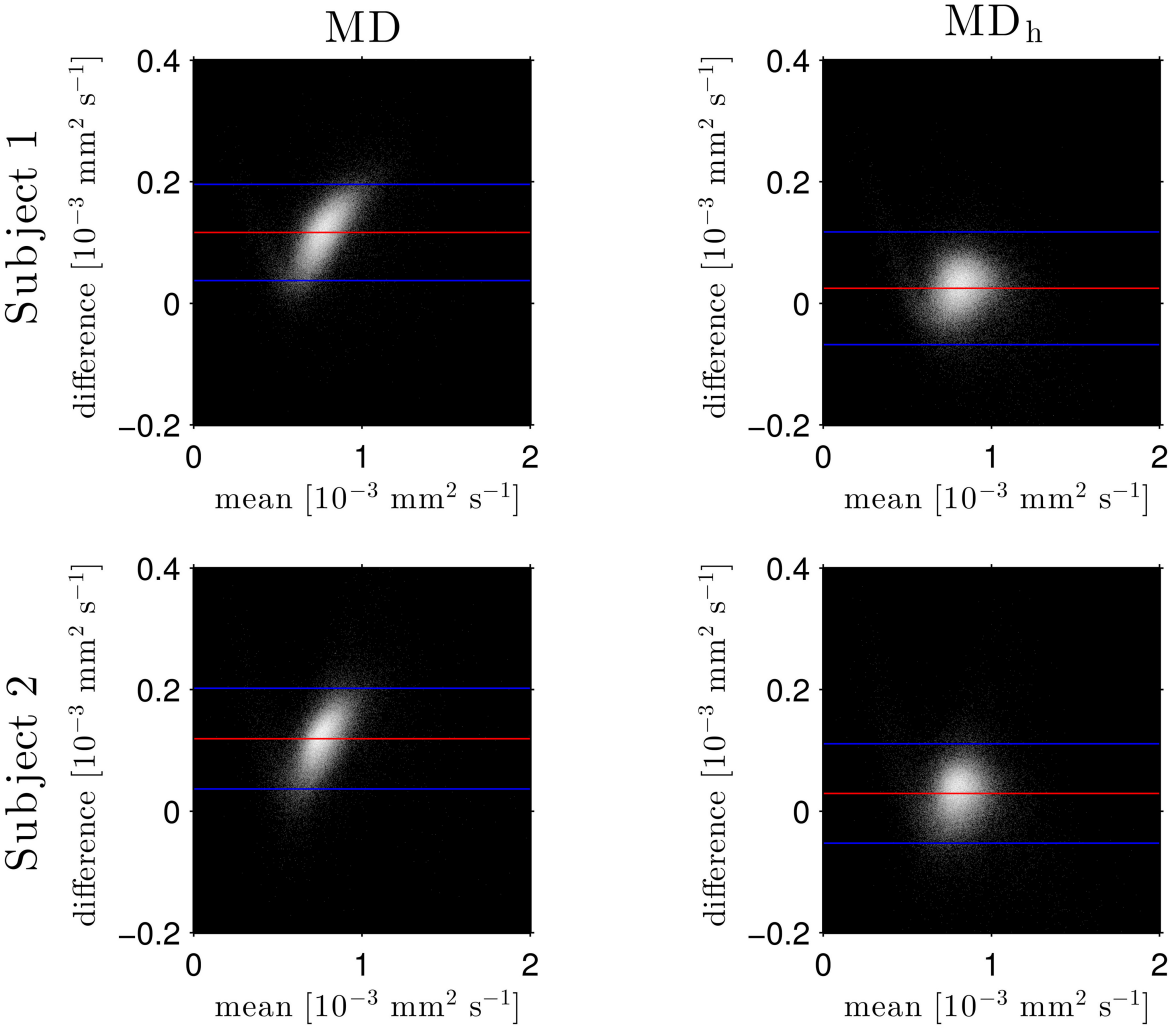
Log density Bland–Altman plots comparing MD_DKI_ estimated via simultaneous fit of the kurtosis tensor and DT using both shells of data, and mean diffusivity from a DT fit of the low-*b*-value shell without (MD, left) and with (MD_h_, right) heuristic diffusional kurtosis correction. Red lines show mean difference, blue lines show ± two standard deviations of the difference (Bland and Altman, 1986). Simultaneous fit of the kurtosis tensor and DT was performed as per Mohammadi et al. (2015). Differences are defined as MD_DKI_ − (MD or MD_h_). Each row shows a different subject as labelled.

**Figure 2:**
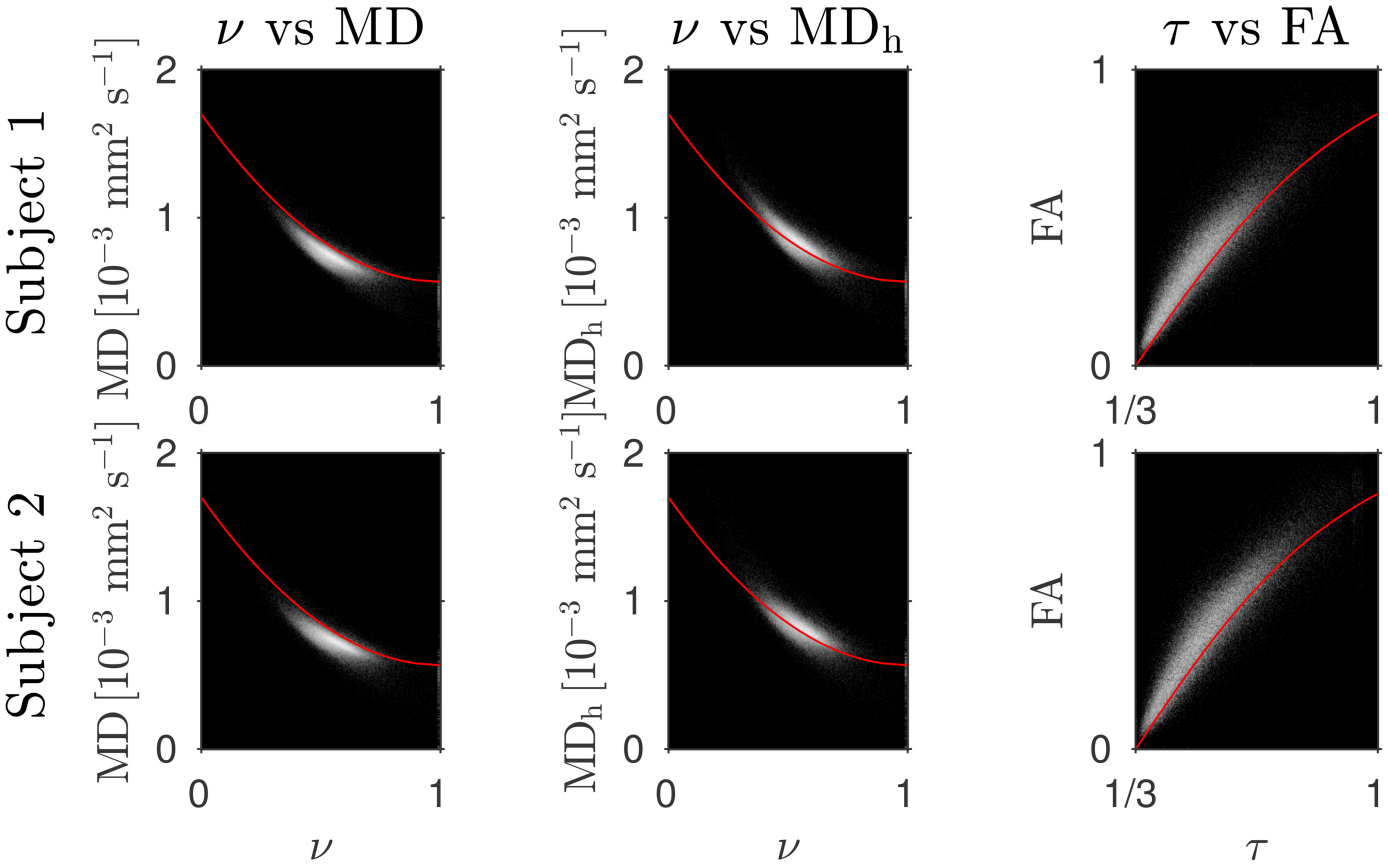
Log density scatter plots comparing DTI invariants (estimated using only the low *b*-value shell of data) and NODDI toolbox fitted parameters (fitted using both shells of data). Each row shows a different subject as labelled. Overlaid red lines in the first and second columns show values of ν for given values of MD and MD_h_, respectively, estimated using Equation (2). The overlaid red lines in the third column show τ estimated using Equation (3) for given FA, with MD set to the mean value in the WM of each subject.

The Bland–Altman plots in Figure 1 show that the heuristically corrected MD, MD_h_, is less biased than the uncorrected MD. The numerical values (mean ± one standard deviation) of the differences in Figure 1 confirm this: for subject 1: 0.117 ± 0.040 (MD), 0.025 ± 0.046 (MD_h_); and for subject 2: 0.119 ± 0.041 (MD), 0.029 ± 0.041 (MD_h_). The effect of this bias on NODDI-DTI estimates is evidenced in Figure 2, where the main bulk of the MD values lies below the red line representing the ν vs. MD prediction of Equation (2). The heuristically corrected values estimated using Equation (5) show much better agreement (Figure 2), implying that we are justified in using Equation (6) to estimate ν. The effect of diffusional kurtosis is much less pronounced for FA in Figure 2, as expected based upon Veraart et al. (2011), implying that we are justified in using Equation (3) to estimate τ.

**Figure 3:**
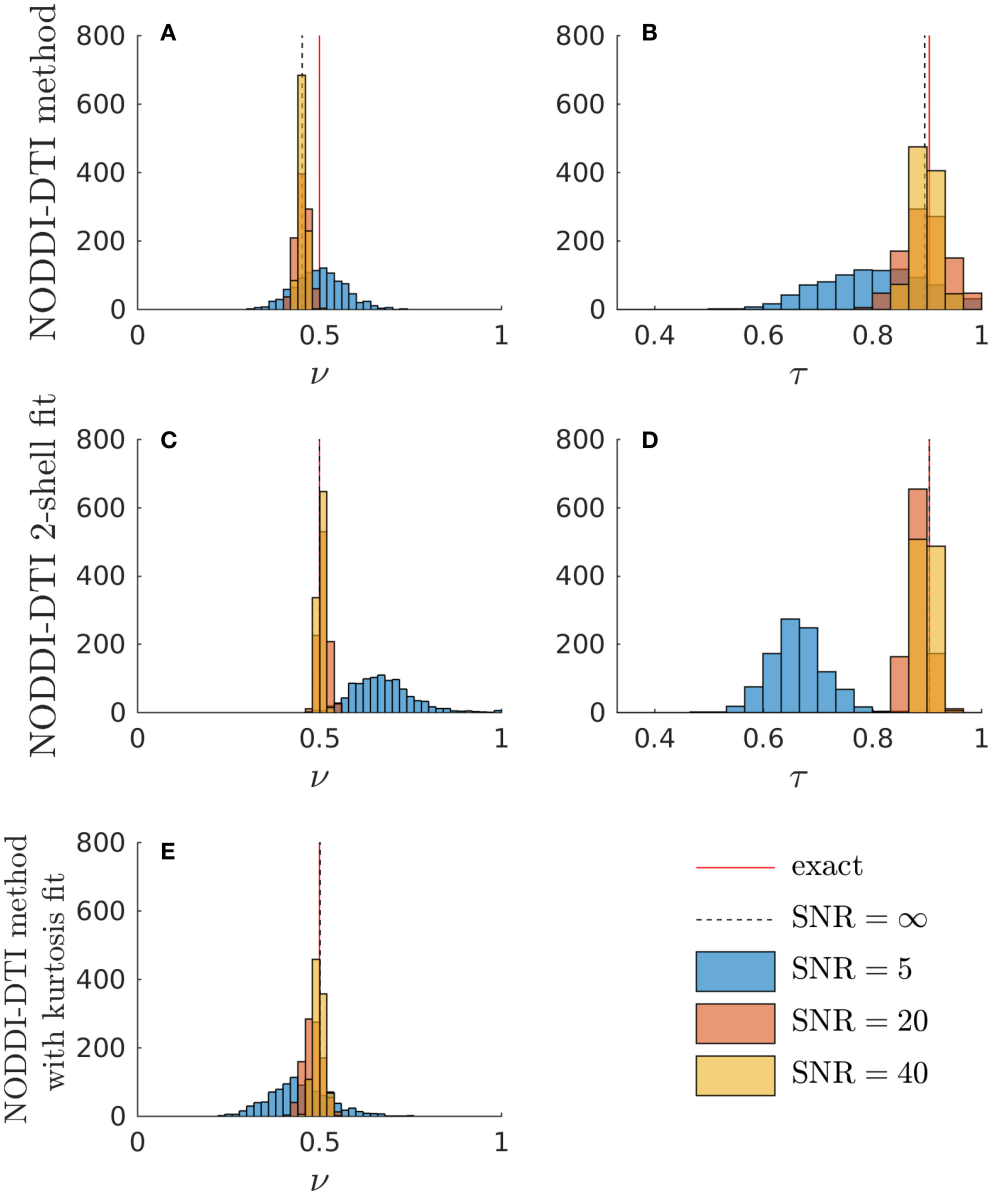
Simulations demonstrating the effect of noise and diffusional kurtosis on NODDI-DTI parameter estimates. Different coloured histograms represent the distribution of the results from repetitions of simulations with different SNR values as given in the legend. In addition, the red line labelled “exact” marks the ground truth value and the dashed black line labelled “SNR = ∞” marks the noise-free result in each case. **(A,B)** were estimated by applying the NODDI-DTI method to the simulated data. **(C,D)** were estimated by fitting the simulated data using the NODDI toolbox. **(E)** was estimated by estimating an MD value from the simulated data that was, through explicit estimation of the diffusional kurtosis contribution, less biased by higher order moments, and then inserting this MD into Equation (2).

Simulations allowed further insight into the behaviour of the NODDI-DTI relations. Shown in Figure 3 are histograms of parameter estimates from data simulated with typical corpus callosum parameters. The ν estimated using Equation (6) showed a bias (Figure 3A). Using an estimate of MD less biased by higher order moments strongly reduced the bias in the ν estimates (Figure 3E), implying that the ν bias in Figure 3A is due to residual diffusional kurtosis effects. The inadequacy of the heuristic diffusional correction for data generated using typical corpus callosum parameters can be explained using previous experimental observations: the corpus callosum is a region of WM where mean diffusional kurtosis is greater than unity (Jensen and Helpern, 2010; Lätt et al., 2013), in contrast to the value of unity assumed in the derivation of Equation (5). Estimates of ν from Equation (2) that did not make any correction for diffusional kurtosis (data not shown) showed a much greater bias, in line with the results shown in Figure 2. At low SNR, the ν estimate from the two-shell fit became biased (Figure 3C), likely because the second shell is strongly affected by noise at low SNR (André et al., 2014). A similar bias can be seen in the ν estimated in parallel with an estimate of diffusional kurtosis (Figure 3E). In contrast, the ν estimated using Equation (6) remained much more robust (Figure 3A). The estimate of τ from Equation (3) showed little bias (Figure 3B), and again the estimates were more robust to noise than for the two-shell fit estimates (Figure 3D). Noise caused unphysical τ estimates when using the NODDI-DTI method: these constituted 1.2% of SNR ≈ 20 estimates and 8.6% of SNR ≈ 5 estimates. There were no unphysical ν estimates.

**Figure 4:**
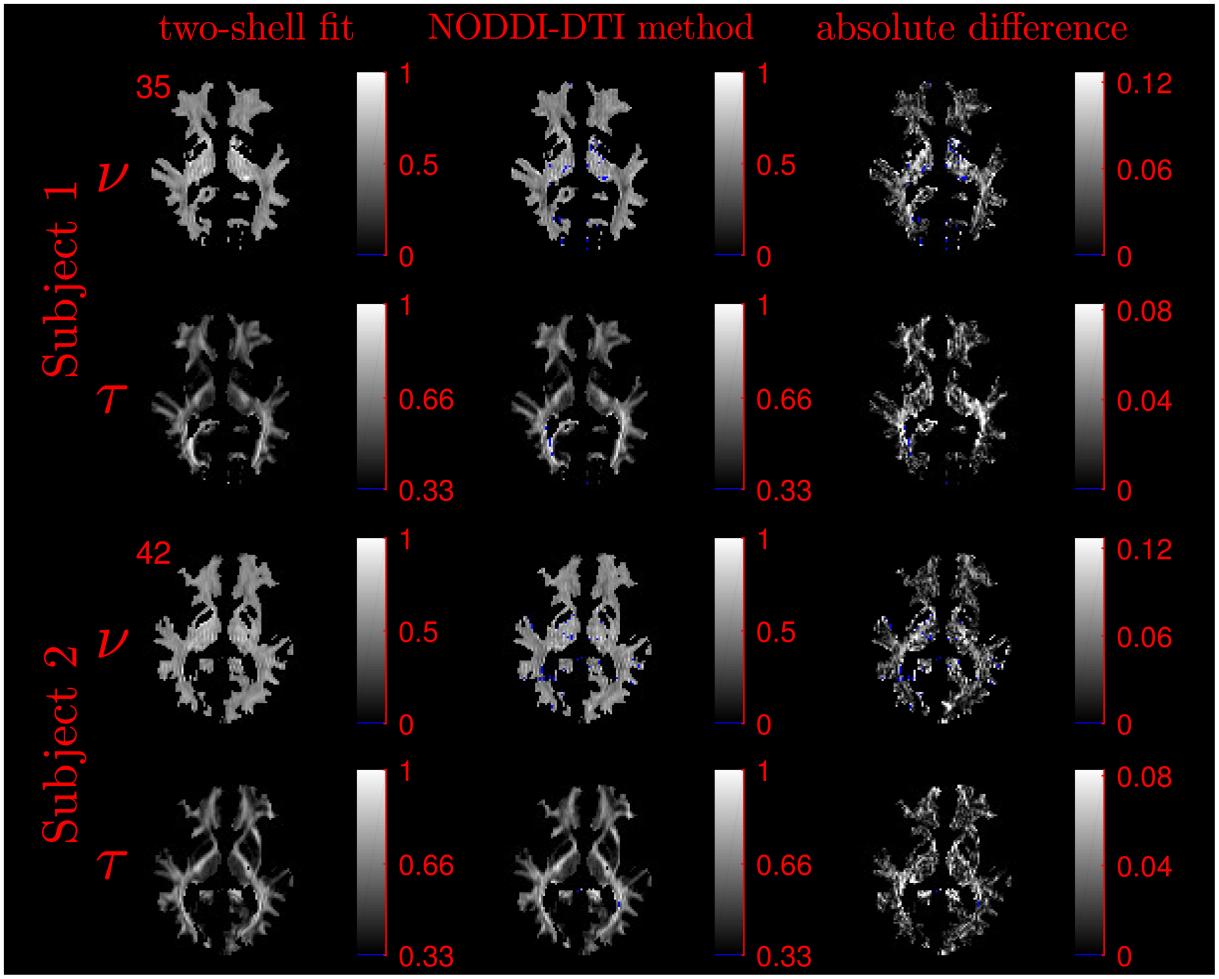
Comparison of maps of parameters estimated using the NODDI-DTI method and fitting two-shells using the NODDI toolbox for subjects 1 and 2. Voxels where the NODDI-DTI method gave an unphysical parameter estimate are shown in blue. Windows are as per the limits of the colour scales beside each map, and the slice number is given at the top left of the row for each subject to allow for cross-referencing with Figures 8, 9.

**Figure 5:**
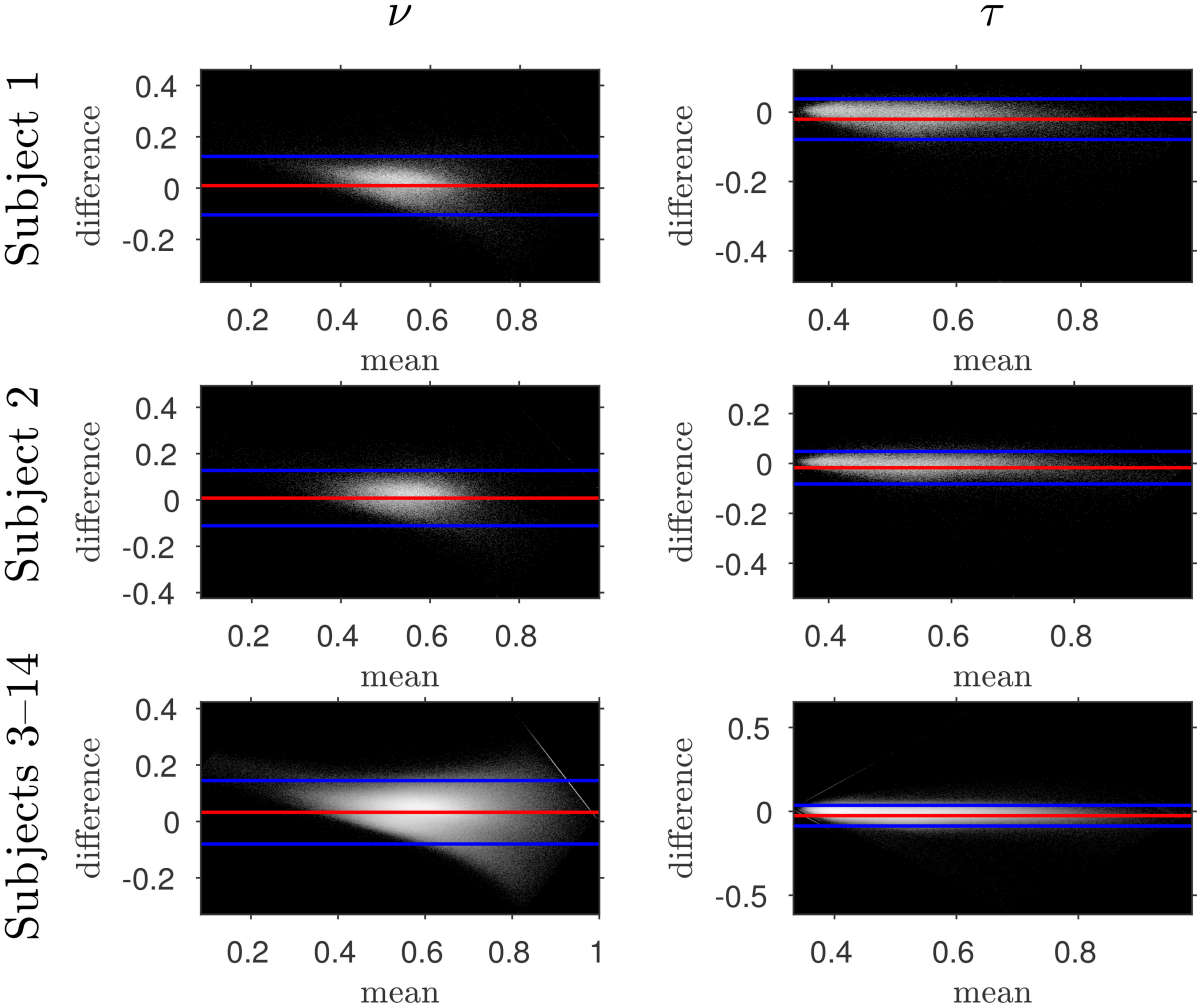
Log density Bland–Altman plots comparing NODDI-DTI-method and two-shell NODDI toolbox fitted results. Red lines show mean difference, blue lines show ± two standard deviations of the difference (Bland and Altman, 1986). Differences are defined as (two-shell NODDI toolbox fitted parameter) − (NODDI-DTI method parameter). Parameter differences for subjects 3–14 have been concatenated into one plot for each parameter. Numerical values of the means and standard deviations of the differences for subjects 1 and 2 are given in Figure 6, and the means and standard deviations of the differences for subjects 3–14 are shown in Figure 7. Axis ranges show bounds of means and differences in each case.

**Figure 6:**
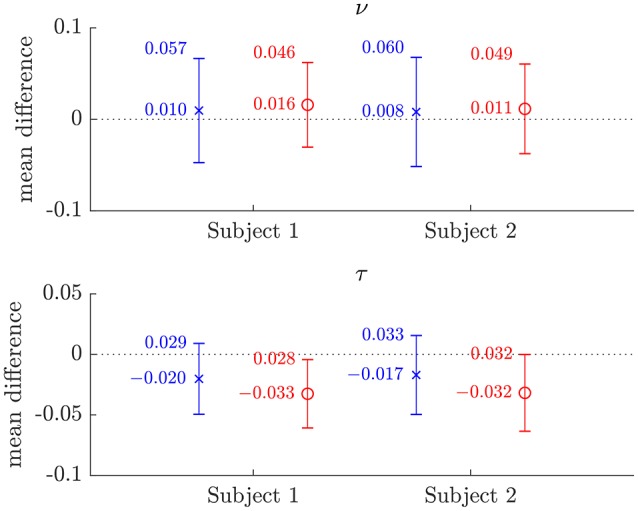
Plots of the mean differences between NODDI-DTI method and two-shell NODDI toolbox fitted (×), and between one-shell NODDI toolbox fitted (using the NODDI toolbox to fit the low *b*-value data) and two-shell NODDI toolbox fitted (o) parameter estimates for subjects 1 and 2. Error bars show ± one standard deviation of the differences. Differences are defined as (two-shell NODDI toolbox fitted parameter) − (estimated parameter). Numerical mean values are given beside each plotted point, and numerical values for the standard deviations are given beside the upper error bar. The dotted lines mark the line of zero bias in each plot.

**Figure 7:**
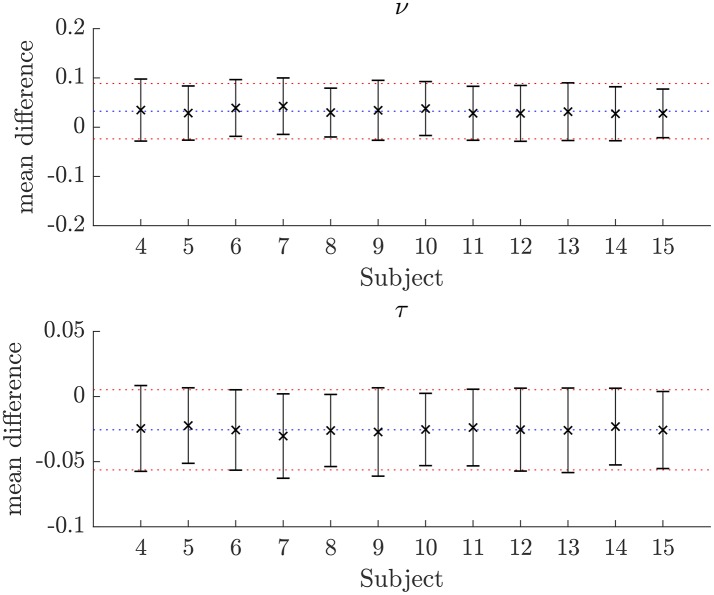
Plots of the mean differences between NODDI-DTI method and two-shell NODDI toolbox fitted parameter estimates for subjects 3–14. Error bars show ± one standard deviation of the differences. Differences are defined as (two-shell NODDI toolbox fitted parameter) − (NODDI-DTI method parameter). The blue dotted lines show the mean difference over the whole group, and the red dotted lines show ± one standard deviation of the differences over the whole group. For ν the mean difference over the group is 0.032 and the standard deviation of the difference is 0.056. For τ the mean difference over the group is −0.026 and the standard deviation of the difference is 0.031.

**Figure 8:**
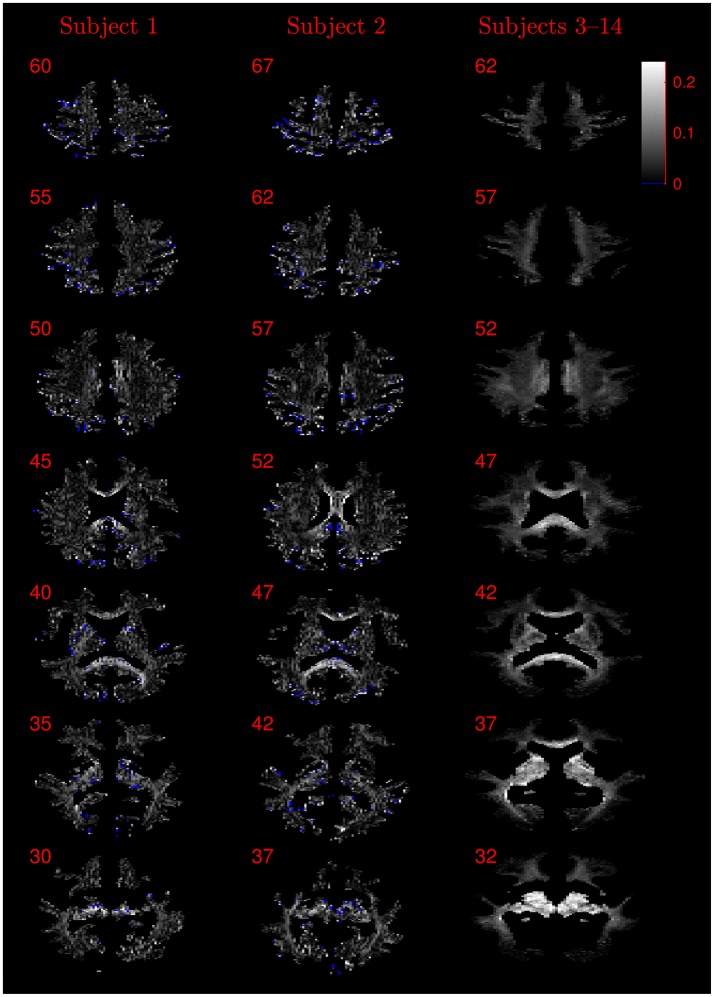
Absolute value maps of the difference between ν estimated by using the NODDI-DTI method and by fitting two-shells with the NODDI toolbox. For subjects 3–14, the absolute value of the mean difference over the spatially normalised images in group space is shown. Slice numbers are given for each row (slice) and column (subject). The extent of the colour scale at the top right shows the windowing for all slices. Blue denotes voxels where the NODDI-DTI method gave an unphysical parameter estimate. Subjects 3–14 show no unphysical parameter estimates here because of the procedure followed to facilitate warping to standard space (see section 2.4). As discussed in the main text, the larger differences visible in the corpus callosum are likely due to incomplete diffusional kurtosis correction, and the larger differences at the base of the brain in subjects 3–14 are likely due to reduced SNR in this region.

**Figure 9:**
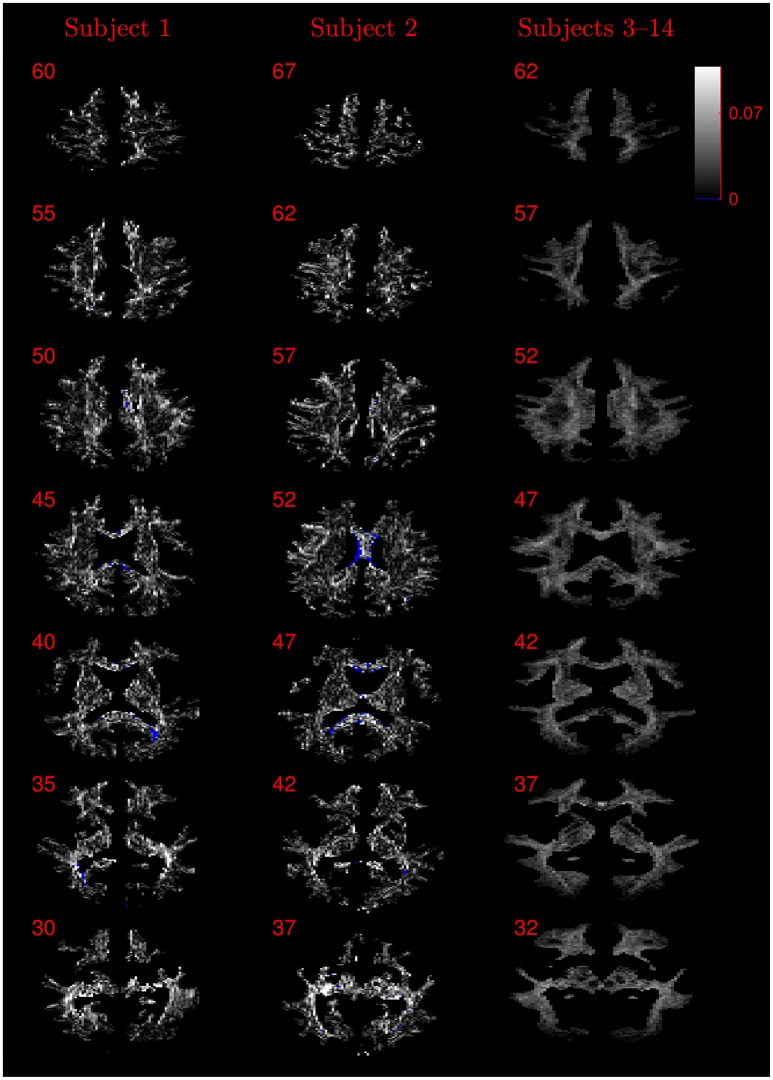
Absolute value maps of the difference between τ estimated by using the NODDI-DTI method and by fitting two-shells with the NODDI toolbox. For subjects 3–14, the absolute value of the mean difference over the spatially normalised images in group space is shown. Slice numbers are given for each row (slice) and column (subject). The extent of the colour scale at the top right shows the windowing for all slices. Blue denotes voxels where the NODDI-DTI method gave an unphysical parameter estimate. Subjects 3–14 show no unphysical parameter estimates here because of the procedure followed to facilitate warping to standard space (see section 2.4).

The similarity of parameters estimated using the NODDI-DTI method to the silver standard results can be seen in Figure 4, which shows parameter maps estimated with each method, along with maps showing the differences between the parameter estimates. Differences between the parameter estimates are further presented in several complementary ways: Bland–Altman plots in Figure 5 show general behaviour, plots of the means and standard deviations of the differences in Figures 6, 7 compare this general behaviour across subjects, and the series of slices in Figures 8, 9 show the behaviour of NODDI-DTI method parameter estimates throughout the WM. The higher ν differences observed in the corpus callosum of all subjects, and at the base of the brain in subjects 3–14 are discussed in the next section. Within protocol comparisons show the best agreement, but all subjects behave similarly, demonstrating the robustness of the NODDI-DTI method. The diagonal line visible to the right of the ν Bland–Altman plot for subjects 3–14 in Figure 5 is due to voxels where the ν estimate of the two-shell fit is estimated to be unity while the NODDI-DTI method gives a more biologically plausible value. The number of such voxels is low, and so they only appear clearly in the combined plot from multiple subjects.

Equations (3) and (6) gave unphysical parameter estimates for some voxels. For subjects 1 and 2 such unphysical ν estimates constituted 2.07% and 2.19% of the total WM voxels respectively; for τ such estimates constituted 0.27% and 0.74%. Subjects 3–14 showed more variation in terms of the numbers of failed voxels, constituting 3.65%–13.39% of WM voxels for ν, and 0.07%–0.37% of WM voxels for τ. Generally, then, the proportion of unphysical parameter estimates was higher for ν than for τ, and a strong variability in the proportion of failed voxels was evident between the different subjects.

The magnitudes of the means and standard deviations of the differences between the NODDI-DTI parameter estimated using a single shell of data and the two-shell NODDI toolbox fits are shown in Figure 6. One-shell NODDI toolbox fitting gave stable fits in this case because the CSF compartment fraction was fixed at zero (Magnollay et al., 2014). The NODDI-DTI method showed smaller mean differences and one-shell NODDI toolbox fitting showed smaller standard deviations of the differences. Overall, however, both methods of parameter estimation were comparable, further demonstrating the validity of the NODDI-DTI method.

## 4. Discussion

This work has demonstrated that, with caveats to be discussed below, parameters of potential neurobiological relevance can be estimated from DTI parameters in healthy white matter using the NODDI-DTI relations, Equations (3) and (6). Correction of the estimated DTI parameters for diffusional kurtosis proved essential in relating these experimental parameters quantitatively to the underlying biology. Importantly, the improved interpretability gained through the NODDI-DTI relations is not only applicable to future DTI studies, but also to existing DTI studies.

As an example of the potential use of the NODDI-DTI method in reinterpreting existing DTI studies, we apply the method superficially to the study of Scholz et al. (2009), who demonstrated a statistically significant FA increase in WM “underlying the intraparietal sulcus” after participants learned to juggle. Assuming no consistent concomitant change in MD (as suggested by the authors not reporting any significant change), this FA increase could be interpreted, using Equation (3), as an increase in τ, i.e., an increase in alignment of neuronal axons in this area with training. This result is much more specific than a change in FA. We note, however, that confirmation of this observation would require reanalysis of the original data, especially since our assumption of no consistent concomitant change in MD may be unwarranted. A follow up study recording multi-shell data, combined with proper mechanistic analysis of the WM plasticity mechanisms, would allow investigation of this effect in more detail.

Unfortunately, the NODDI-DTI method did not always give physically plausible parameter estimates for the datasets studied herein. We posit four overlapping explanations for these unphysical estimates, related to NODDI-DTI assumptions.

Assumption 1: *DTI parameters can be accurately estimated from the diffusion signal*. Errors in DTI parameter estimation will lead to errors in parameters estimated using Equations (3) and (6), potentially giving unphysical parameter estimates. The *b*-values used likely resulted in overestimates of FA in regions of high anisotropy due to poor estimates of the smallest DT eigenvalue (Pierpaoli et al., 1996; Jones and Basser, 2004), explaining why many of the unphysical τ estimates were found in the highly anisotropic corpus callosum (Figure 9).

It should be noted that while noise can give rise to bias in the estimated DTI parameters, and thus the estimated biophysical parameters, Figure 3 demonstrates that in low SNR conditions the reliance of the NODDI-DTI method on only the first shell of data makes it more robust than two-shell based approaches. As SNR varies across the WM of the brain in most practical acquisitions, this implies that the differences observed relative to the two-shell fit could, in some regions, be due to noise adversely affecting the second shell of data, and so biasing the parameters estimated using the NODDI toolbox two-shell fitting procedure. This explains the greater ν differences for subjects 3–14 (Figure 8): the smaller voxel size decreases SNR, causing bias and increased variance in the NODDI toolbox-fitted and NODDI-DTI method-estimated parameters. This effect is seen to be especially prominent in the WM at the base of the brain, where SNR is low due to the comparatively large distance from the receiver coil elements (Wiggins et al., 2006) and the strong influence of physiological noise sources (Brooks et al., 2013; Sclocco et al., 2017).

Assumption 2: *CSF can be ignored in voxels with a high probability of being WM*. NODDI-DTI could give unphysical parameter estimates whenever a voxel contains a significant amount of CSF: the high diffusivity of CSF (Zhang et al., 2012) can take MD_h_ outside the limits of NODDI-DTI (Equation 4). Figures 8, 9 show that many of the voxels where NODDI-DTI gave unphysical parameter estimates are close to the edge of the WM mask, in line with partial volume effects being important. Because CSF volume fraction was fixed at zero in our NODDI toolbox fits, residual partial volume effects may also have affected those parameter estimates. The variability in the number of unphysical parameter estimates evidenced by the group of subjects 3–14 likely reflects the variable success of our method of thresholding for determining which voxels are WM voxels.

Assumption 3: MD *can be heuristically corrected for diffusional kurtosis bias*. Figures 1–3 show that diffusional kurtosis affects our estimates of MD; such effects could take MD estimates out of the range of applicability for NODDI-DTI. While our heuristic correction (Equation 5) substantially mitigates this issue (Figure 2), it does not completely eliminate it (Figure 3). This is evident in the ν Bland–Altman plots (Figure 5), where the mean and standard deviation of the differences visibly vary with the mean ν estimate, implying (via Equation 6) residual correlation between the errors and the corrected MD.

Residual bias in the region around the corpus callosum of the ν difference maps in Figure 8 can be explained by incomplete diffusional kurtosis correction. Here, the mean diffusional kurtosis is greater than unity (Jensen and Helpern, 2010; Lätt et al., 2013), and the low DT eigenvalues are poorly estimated (see Assumption 1), meaning that the assumptions of the heuristic correction are not met. Simulations using corpus callosum-like ν and τ parameters showed that using a less-biased estimate of the MD in Equation (2) could improve the ν estimate substantially (Figure 3E).

Assumption 4: *The NODDI-DTI signal model is a valid representation of the diffusion signal*. Several criticisms have been levelled in the literature against assumptions made by the NODDI model (Zhang et al., 2012). Because the NODDI-DTI model inherits these assumptions, these criticisms also apply to the NODDI-DTI model:

1. WM voxels containing perpendicularly crossing fibre bundles cannot formally be represented (Jeurissen et al., 2013),
2. The tortuosity model constraining the extraneurite diffusivity may be unrealistic (Jelescu et al., 2016; Kaden et al., 2016), though swapping the NODDI tortuosity model from Zhang et al. (2012) for that given by Kaden et al. (2016) would engender no changes to Equations (2) and (3),
3. The value of *d*, far from being constant as assumed by NODDI-DTI, varies throughout the brain (Kaden et al., 2016), and between the intra- and extra-neurite compartments (Jelescu et al., 2016).

Unphysical parameter estimates are likely to arise when applying the NODDI-DTI method to voxels where the NODDI-DTI signal model is not a valid representation. Such failures are not immediately apparent in the NODDI toolbox fitted parameters because constraints in the fitting procedure mean parameters outside the physical range can never be returned, regardless of whether the model is biologically plausible for a given voxel. Examples of biologically implausible parameter estimates being returned by the fitting procedure are voxels estimated to have ν ≈ 1, which give rise to the stripe evident in Figure 5 for the combined data from subjects 3–14. It is beyond the scope of this article to examine these problems in more depth; we simply emphasise here that one must take stock of the assumptions of a model before placing too much emphasis on interpreting the results of applying it.

The greater number of unphysical NODDI-DTI method parameter estimates for ν as compared to τ can be explained by ν estimation being more sensitive to partial volume and diffusional kurtosis effects. This is borne out by the locations of the failures (Figure 8): mainly either close to the edge of the WM mask (implying partial volume effects), or in regions of high anisotropy (implying residual diffusional kurtosis effects).

Pathology could further undermine the assumptions underlying NODDI-DTI: pathological processes can lead to free water located far from CSF compartments (Pasternak et al., 2009), can affect mean kurtosis values (Guglielmetti et al., 2016), and could affect the “true” value of *d* (Jelescu et al., 2015). We thus recommend that NODDI-DTI not be applied to non-healthy appearing WM without further adaptation and validation. It should be noted that ageing can also lead to changes in mean kurtosis (Falangola et al., 2008), implying a need to vary the fixed value used for mean diffusional kurtosis in the heuristic correction (see Appendix B) in order to apply this correction to older subjects; this is the subject of future work.

In this work, the NODDI-DTI method was only applied to WM. We refrained from applying the method to other brain tissues, e.g., cortical grey matter, as recent research has suggested that the underlying NODDI assumptions need to be modified for different tissue types (Kaden et al., 2016). Further development and validation is thus needed before applying NODDI-DTI in brain tissue other than WM.

NODDI-DTI could be improved and made more appropriate for clinical studies through investigation of the following points. Unphysical parameter estimates could be pragmatically eliminated by constraining DT fitting using Equations (4) (appropriately corrected using Equation 5). The lack of a CSF volume fraction could potentially be mitigated without requiring extra data acquisition by incorporating the free water elimination method (Pasternak et al., 2009; Metzler-Baddeley et al., 2012; van Bruggen et al., 2013) into NODDI-DTI. Use of more stringent thresholds for determining which voxels are classified as WM and improved tissue segmentation procedures (Lorio et al., 2016) could also limit the influence of partial volume effects. Known values of mean diffusional kurtosis in WM (Jensen and Helpern, 2010; Lätt et al., 2013; André et al., 2014; Mohammadi et al., 2015) could be used to construct mean diffusional kurtosis Bayesian priors (Taquet et al., 2015; Alexander et al., 2017), or diffusional kurtosis corrected MD and FA could be measured directly using time-efficient methods (Hansen et al., 2016). Importantly, the latter scheme also provides a mean diffusional kurtosis parameter which could, in principle, allow the NODDI-DTI assumptions to be relaxed by allowing estimation of a further biophysical parameter, e.g., *d* (Kaden et al., 2016) or the CSF volume fraction (Zhang et al., 2012).

We finish by providing practical recommendations for a minimal NODDI-DTI acquisition scheme. Results at *b* = 1, 000 s mm^2^ were reasonable, and so we would recommend this as a lower *b*-value bound. An upper bound on *b*-value comes from ensuring diffusional kurtosis does not constitute the majority of the diffusion contrast. Equation B.3 in Appendix B shows that [assuming the apparent diffusional kurtosis is approximately unity (Jensen and Helpern, 2010; André et al., 2014; Mohammadi et al., 2015)] choosing *b*≪6/*d* ≈ 3, 500 s mm^2^ means that the DT dominates diffusion contrast, giving an upper *b*-value bound. High resolution acquisitions which maintain sufficient SNR for estimation of DT parameters (Jones and Basser, 2004) are recommended to reduce partial volume effects, though decreases in voxel size should not come at the expense of too much loss of SNR. Accurate DT estimation requires measurement of at least 30 distinct diffusion directions (Jones, 2014); we recommend at least this number for application of NODDI-DTI, although the lowest number of orientations tested here was 60.

## 5. Conclusions

We have estimated biophysical parameters representing neurite density and dispersion with reasonable accuracy from diffusion tensor parameters estimated from single-shell diffusion data. Heuristic kurtosis correction of MD was necessary to remove diffusional kurtosis bias; use of corrections such as that derived here could improve other analyses of single-shell diffusion data requiring quantitative MD estimates.

NODDI-DTI potentially opens up two new opportunities: (a) more specific neurobiological interpretation of observed microstructural changes in DTI data (including interpretation of existing datasets), and (b) simple and time efficient estimation of biophysical parameters from smaller diffusion datasets, despite limitations due to the underlying model and difficulties estimating accurate diffusion tensors.

## Author contributions

LE and SM developed the initial idea, and acquired and analysed the data. IE and SM acquired and preprocessed the data for the group of 12 subjects. LE wrote the manuscript and developed the theory, with substantial critical revisions for intellectual content and interpretation of the data provided by all authors.

### Conflict of interest statement

The authors declare that the research was conducted in the absence of any commercial or financial relationships that could be construed as a potential conflict of interest.
