# Supplementary material for: NODDI-DTI: Estimating Neurite Orientation and Dispersion Parameters from a Diffusion Tensor in Healthy White Matter
Source: Front Neurosci. 2017 Dec 20;11:720. doi: 10.3389/fnins.2017.00720 (PMC5742359; doi:10.3389/fnins.2017.00720)
Supplement: Supplementary file 1 [file DataSheet1.pdf]

Appendix to “NODDI-DTI: Estimating Neurite Orientation and Dispersion Parameters from a Diffusion Tensor in Healthy White Matter”†

Luke J. Edwards[1,2,*], **Kerrin J. Pine**[1,2], **Isabel Ellerbrock**[3], **Nikolaus Weiskopf**[1,2] and **Siawoosh Mohammadi**[1,2,3]
*Correspondence:
Luke J. Edwards, MPI-CBS, Stephanstr. 1a, 04103 Leipzig, Germany
ledwards@cbs.mpg.de

A DERIVATION OF NODDI-DTI RELATIONS

A.1 Diffusion tensor of the NODDI-DTI signal model

We begin our derivation of the NODDI-DTI relations by deriving the DT arising from the NODDI-DTI signal model. This derivation is similar to that of Jespersen et al. (2012), where the DT of a precursor to the NODDI-DTI model (Zhang et al., 2011) was derived.

The normalised signal arising from the NODDI-DTI signal model can be written (Zhang et al., 2012)

$$S = \nu \int p(\kappa, \vec{\mu}, \vec{n}) \exp\{-bd\vec{q}^{\,\mathrm{t}}\vec{n}\vec{n}^{\,\mathrm{t}}\vec{q}\}\,\mathrm{d}\vec{n} + (1-\nu)\exp\{-b\vec{q}^{\,\mathrm{t}}D_{\mathrm{ec}}\vec{q}\}, \tag{A.1}$$

where the first term represents the intraneurite water compartment with diffusivity d parallel to the neurite and zero perpendicular to it; the second term represents the extraneurite water compartment; arrows denote normalised vectors; $\cdot^{\mathrm{t}}$ denotes transposition; $\vec{q}$ is the diffusion gradient vector; ν represents neurite density; and

$$D_{\mathrm{ec}} = d \int p(\kappa, \vec{\mu}, \vec{n}) \left(\vec{n}\vec{n}^{\,\mathrm{t}} + (1-\nu)(\mathbf{1} - \vec{n}\vec{n}^{\,\mathrm{t}})\right) \mathrm{d}\vec{n}, \tag{A.2}$$

the DT of the extraneurite compartment, where $\mathbf{1}$ is the 3×3 unit matrix. The form of the extraneurite DT arises from assuming that: the diffusivity of the extraneurite space in the absence of neurites is equal to the intraneurite diffusivity along the direction of the neurite (Zhang et al., 2012), the neurites reduce the diffusivity in a long-time-limit tortuous manner (Zhang et al., 2012), and extracellular water is in fast exchange among all neurite orientations (Kaden et al., 2016). The probability density

$$p(\kappa, \vec{\mu}, \vec{n}) = \frac{\exp\{-\kappa(\vec{\mu}^{\,\mathrm{t}}\vec{n})^2\}}{\int \exp\{-\kappa(\vec{\mu}^{\,\mathrm{t}}\vec{m})^2\}\,\mathrm{d}\vec{m}} \tag{A.3}$$

is a Watson distribution giving the distribution of neurites about a main orientation $\vec{\mu}$ with dispersion parameter κ (Zhang et al., 2012). Isotropically distributed neurites correspond to $\kappa = 0$, neurites perfectly aligned along $\vec{\mu}$ correspond to $\kappa \to \infty$.

†Edwards et al. (2017)

Equation (A.1) can be equated with an expansion of the normalised diffusion signal in b (Jensen et al., 2005),

$$\log(S) = -b\vec{q}^{\,\mathrm{t}} D_{\mathrm{NODDI-DTI}}\vec{q} + O(b^2), \tag{A.4}$$

such that the DT can be extracted by inspection from

$$\left.\frac{\mathrm{d}\log(S)}{\mathrm{d}b}\right|_{b=0} = -\nu d \int p(\kappa, \vec{\mu}, \vec{n}) \vec{q}^{\,\mathrm{t}} \vec{n}\vec{n}^{\,\mathrm{t}} \vec{q}\, \mathrm{d}\vec{n} - (1-\nu)\vec{q}^{\,\mathrm{t}} D_{\mathrm{ec}}\vec{q} \tag{A.5}$$

$$= -\vec{q}^{\,\mathrm{t}} \left[d \int p(\kappa, \vec{\mu}, \vec{n}) \left(\vec{n}\vec{n}^{\,\mathrm{t}} + (1-\nu)^2(\mathbf{1} - \vec{n}\vec{n}^{\,\mathrm{t}}) \right) \mathrm{d}\vec{n} \right] \vec{q} \tag{A.6}$$

as

$$D_{\mathrm{NODDI-DTI}} = d \int p(\kappa, \vec{\mu}, \vec{n}) \left(\vec{n}\vec{n}^{\,\mathrm{t}} + (1-\nu)^2(\mathbf{1} - \vec{n}\vec{n}^{\,\mathrm{t}}) \right) \mathrm{d}\vec{n} \tag{A.7}$$

$$= (1-\nu)^2 d\mathbf{1} + (2-\nu)\nu d \int p(\kappa, \vec{\mu}, \vec{n}) \vec{n}\vec{n}^{\,\mathrm{t}}\, \mathrm{d}\vec{n}\,. \tag{A.8}$$

The integral appearing on the right-hand side of Equation (A.8) is given by (Jespersen et al., 2012)

$$\int p(\kappa, \vec{\mu}, \vec{n}) \vec{n}\vec{n}^{\,\mathrm{t}}\, \mathrm{d}\vec{n} = \tau \vec{\mu}\vec{\mu}^{\,\mathrm{t}} + \frac{(1-\tau)}{2}(\mathbf{1} - \vec{\mu}\vec{\mu}^{\,\mathrm{t}}), \tag{A.9}$$

where τ is defined in Equation (1). Inserting Equation (A.9) into Equation (A.8) gives

$$D_{\mathrm{NODDI-DTI}} = ((1-\nu)^2 d + (2-\nu)\nu\tau d)\vec{\mu}\vec{\mu}^{\,\mathrm{t}} + ((1-\nu)^2 d + (2-\nu)\nu\frac{(1-\tau)}{2} d)(\mathbf{1} - \vec{\mu}\vec{\mu}^{\,\mathrm{t}}), \tag{A.10}$$

from which, by inspection, the largest eigenvalue (corresponding to an eigenvector co-linear with the main neurite orientation) is

$$\lambda_1 = \vec{\mu}^{\,\mathrm{t}} D_{\mathrm{NODDI-DTI}}\, \vec{\mu} = (1-\nu)^2 d + (2-\nu)\nu\tau d, \tag{A.11}$$

and the other two eigenvalues are degenerate (with respective eigenvectors arbitrarily defined in the plane perpendicular to $\vec{\mu}$):

$$\lambda_2 = \lambda_3 = (1-\nu)^2 d + (2-\nu)\nu\frac{(1-\tau)}{2} d. \tag{A.12}$$

Because $\lambda_1 \geq \lambda_2$ for $\tau > 1/3$, when the primary eigenvector of $D_{\mathrm{NODDI-DTI}}$ is well-defined (i.e. when $D_{\mathrm{NODDI-DTI}}$ is not isotropic), this eigenvector is formally equivalent to the main neurite orientation, as previously observed empirically (Daducci et al., 2015).

A.2 Relation of ν to MD

MD is defined in terms of the eigenvalues of a DT as (Jones, 2014)

$$\mathrm{MD} = \frac{\lambda_1 + \lambda_2 + \lambda_3}{3}. \tag{A.13}$$

Inserting the eigenvalues from Equations (A.11) and (A.12) results in

$$\frac{3\mathrm{MD}}{d} = (2-\nu)\nu + 3(1-\nu)^2. \tag{A.14}$$

Upon solving this quadratic equation for ν, one obtains

$$\nu = 1 \pm \sqrt{\frac{1}{2}\left(\frac{3\mathrm{MD}}{d} - 1\right)}, \tag{A.15}$$

where the sign ambiguity is resolved by recalling that $\nu \leq 1$, giving Equation (2).

A.3 Relation of τ to MD and FA

A convenient definition of FA in terms of the eigenvalues of a DT is (Jones, 2014):

$$\mathrm{FA} = \sqrt{\frac{3}{2}}\sqrt{\frac{(\lambda_1-\mathrm{MD})^2 + (\lambda_2-\mathrm{MD})^2 + (\lambda_3-\mathrm{MD})^2}{\lambda_1^2+\lambda_2^2+\lambda_3^2}}. \tag{A.16}$$

Because the eigenvalues are linear functions of τ (Equations (A.11) and (A.12)) and there is symmetry between them, it is convenient to simplify this equation by solving for λ_2 before proceeding further. Utilising the identities $\lambda_1 = 3\mathrm{MD} - \lambda_2 - \lambda_3$ (Equation (A.13)) and $\lambda_2 = \lambda_3$ (Equation (A.12)), Equation (A.16) becomes:

$$\mathrm{FA}^2 = \frac{9(\lambda_2-\mathrm{MD})^2}{(3\mathrm{MD}-2\lambda_2)^2 + 2\lambda_2^2}, \tag{A.17}$$

which can be rearranged into the quadratic equation

$$\lambda_2^2 - 2\mathrm{MD}\lambda_2 + 3\mathrm{MD}^2\frac{1-\mathrm{FA}^2}{3-2\mathrm{FA}^2} = 0 \tag{A.18}$$

for which the solutions are:

$$\lambda_2 = \mathrm{MD}\left(1 \pm \frac{\mathrm{FA}}{\sqrt{3-2\mathrm{FA}^2}}\right). \tag{A.19}$$

Equation (A.19), reveals that all we must do to express τ in terms of MD and FA is (i) express τ in terms of MD and λ_2 and then (ii) substitute Equation (A.19) into the resulting expression.

Part (i) is achieved by substituting Equation (2) into Equation (A.12), then simplifying to give

$$\lambda_2 = \frac{1}{4}(d + 3\mathrm{MD} - 3(d-\mathrm{MD})\tau), \tag{A.20}$$

which, after rearranging for τ, reveals

$$\tau = \frac{1}{3}\left(\frac{1}{d-\mathrm{MD}}(d + 3\mathrm{MD} - 4\lambda_2)\right). \tag{A.21}$$

We can now perform part (ii): inserting Equation (A.19) into Equation (A.21) and simplifying gives the result

$$\tau = \frac{1}{3}\left(1 \mp \frac{4}{d - \mathrm{MD}}\frac{\mathrm{MD}\cdot\mathrm{FA}}{\sqrt{3-2\mathrm{FA}^2}}\right). \tag{A.22}$$

The sign ambiguity is resolved by recalling that $\tau \geq 1/3$ (Jelescu et al., 2015) and that both MD and FA are nonnegative, resulting in Equation (3).

Equation (3) is ill-defined at MD $= d$ (the denominator of the second term goes to zero); we classify values at this point as 'unphysical' unless FA is also zero. This latter situation corresponds to the complete absence of fibres (as confirmed by inserting MD $= d$ into Equation (2)), and so τ is taken to equal its isotropic value, 1/3.

B HEURISTIC CORRECTION OF MD FOR DIFFUSIONAL KURTOSIS

When diffusional kurtosis and higher order moments are zero, the normalised diffusion signal S is related to the apparent diffusivity, D_{app}, by (Basser et al., 1994)

$$\log(S) = -bD_{\mathrm{app}}, \tag{B.1}$$

giving

$$\mathrm{MD} = \langle D_{\mathrm{app}}\rangle = \frac{\langle\log(S)\rangle}{-b}, \tag{B.2}$$

where $\langle\cdot\rangle$ denotes averaging over all diffusion directions.

The complicated microstructure of white matter requires higher order moments to represent the diffusion signal (Jensen et al., 2005; Jensen and Helpern, 2010; Veraart et al., 2011). To the order of the diffusional kurtosis the normalised diffusion signal is

$$\log(S) = -bD_{\mathrm{app}} + \frac{b^2}{6}{D_{\mathrm{app}}}^2 K_{\mathrm{app}}, \tag{B.3}$$

where K_{app} is the apparent diffusional kurtosis (Jensen et al., 2005). The effective mean diffusivity $\mathrm{MD}_{\mathrm{eff}}$ derived from this signal (as per Equation (B.2)) would be

$$\mathrm{MD}_{\mathrm{eff}} = \frac{\langle\log(S)\rangle}{-b} = \mathrm{MD} - \frac{b}{6}\left\langle {D_{\mathrm{app}}}^2 K_{\mathrm{app}}\right\rangle, \tag{B.4}$$

which differs from the true MD by a term which we call 'diffusional kurtosis bias'. While good estimates of unbiased MD can be obtained from multi-b-value data (Veraart et al., 2011), such extra data is not available for most DTI acquisitions, and so we derive and use an heuristic correction to mitigate diffusional kurtosis bias.

Defining the covariance of ${D_{\mathrm{app}}}^2$ and K_{app}:

$$\mathrm{cov}\left({D_{\mathrm{app}}}^2, K_{\mathrm{app}}\right) = \left\langle\left({D_{\mathrm{app}}}^2 - \left\langle {D_{\mathrm{app}}}^2\right\rangle\right)(K_{\mathrm{app}} - \mathrm{MK})\right\rangle \tag{B.5}$$

$$= \left\langle {D_{\mathrm{app}}}^2 K_{\mathrm{app}}\right\rangle - \left\langle {D_{\mathrm{app}}}^2\right\rangle \mathrm{MK}, \tag{B.6}$$

where $\mathrm{MK} = \langle K_{\mathrm{app}} \rangle$, the mean kurtosis, we can write Equation (B.4) in the form

$$\mathrm{MD}_{\mathrm{eff}} = \mathrm{MD} - \frac{b}{6}\left(\left\langle {D_{\mathrm{app}}}^2 \right\rangle \mathrm{MK} + \mathrm{cov}\left({D_{\mathrm{app}}}^2, K_{\mathrm{app}}\right)\right). \tag{B.7}$$

We pragmatically assert that $\mathrm{cov}\left({D_{\mathrm{app}}}^2, K_{\mathrm{app}}\right) = 0$, i.e. we assume that the squared apparent diffusivity and apparent diffusional kurtosis are uncorrelated. This assertion results in

$$\mathrm{MD}_{\mathrm{eff}} \approx \mathrm{MD} - \frac{b}{6}\left\langle {D_{\mathrm{app}}}^2 \right\rangle \mathrm{MK}. \tag{B.8}$$

To compute the average in Equation (B.8), we express D_{app} in components of the DT, D, and orientation vector, $\vec{q}$, i.e. (Jensen and Helpern, 2010)

$$\left\langle {D_{\mathrm{app}}}^2 \right\rangle = \sum_{i,j,k,l=1}^{3} \langle q_i q_j q_k q_l D_{ij} D_{kl} \rangle. \tag{B.9}$$

Because we integrate over all $\vec{q}$ on the sphere, we can freely choose the basis of $\vec{q}$. We thus choose the diagonal basis of D, simplifying Equation (B.9) to:

$$\left\langle {D_{\mathrm{app}}}^2 \right\rangle = \sum_{i,k=1}^{3} \left\langle q_i^2 q_k^2 \lambda_i \lambda_k \right\rangle = \sum_{i,k=1}^{3} \left\langle q_i^2 q_k^2 \right\rangle \lambda_i \lambda_k, \tag{B.10}$$

where λ_i is the ith eigenvalue of D and is independent of orientation. Averages over the products of the components q_i evaluate to $\left\langle q_i^2 q_k^2 \right\rangle = (1 + 2\delta_{ik})/15$, where δ_{ik} is the Kronecker delta, thus

$$\left\langle {D_{\mathrm{app}}}^2 \right\rangle = \sum_{i,k=1}^{3} \frac{1 + 2\delta_{ik}}{15} \lambda_i \lambda_k. \tag{B.11}$$

Inserting Equation (B.11) into Equation (B.8) and rearranging gives:

$$\mathrm{MD} \approx \mathrm{MD}_{\mathrm{eff}} + \frac{b}{6}\left(\sum_{i,k=1}^{3} \frac{1 + 2\delta_{ik}}{15} \lambda_i \lambda_k\right) \mathrm{MK}. \tag{B.12}$$

This approximation becomes independent of diffusional kurtosis upon making two further assumptions: 1) the measured (diffusional kurtosis biased) eigenvalues can be substituted for the 'true' eigenvalues; and 2) MK = 1, as found empirically in much healthy WM (Jensen and Helpern, 2010; Lätt et al., 2013; André et al., 2014; Mohammadi et al., 2015). These two assumptions result in the heuristically corrected MD of Equation (5).

REFERENCES

André, E.D., Grinberg, F., Farrher, E., Maximov, I.I., Shah, N.J., Meyer, C., Jaspar, M., Muto, V., Phillips, C., Balteau, E., 2014. Influence of noise correction on intra- and inter-subject variability of quantitative metrics in diffusion kurtosis imaging. PLoS ONE 9, 1–15. doi:10.1371/journal.pone.0094531.

Basser, P.J., Mattiello, J., LeBihan, D., 1994. MR diffusion tensor spectroscopy and imaging. Biophysical Journal 66, 259–267. doi:10.1016/S0006-3495(94)80775-1.

Daducci, A., Canales-Rodríguez, E.J., Zhang, H., Dyrby, T.B., Alexander, D.C., Thiran, J.P., 2015. Accelerated microstructure imaging via convex optimization (AMICO) from diffusion MRI data. NeuroImage 105, 32–44. doi:10.1016/j.neuroimage.2014.10.026.

Edwards, L.J., Pine, K.J., Ellerbrock, I., Weiskopf, N., Mohammadi, S., 2017. NODDI-DTI: Estimating neurite orientation and dispersion parameters from a diffusion tensor in healthy white matter. Front. Neurosci. doi:10.3389/fnins.2017.00720.

Jelescu, I.O., Veraart, J., Adisetiyo, V., Milla, S.S., Novikov, D.S., Fieremans, E., 2015. One diffusion acquisition and different white matter models: How does microstructure change in human early development based on WMTI and NODDI? NeuroImage 107, 242–256. doi:10.1016/j.neuroimage.2014.12.009.

Jensen, J.H., Helpern, J.A., 2010. MRI quantification of non-gaussian water diffusion by kurtosis analysis. NMR in Biomedicine 23, 698–710. doi:10.1002/nbm.1518.

Jensen, J.H., Helpern, J.A., Ramani, A., Lu, H., Kaczynski, K., 2005. Diffusional kurtosis imaging: The quantification of non-gaussian water diffusion by means of magnetic resonance imaging. Magnetic Resonance in Medicine 53, 1432–1440. doi:10.1002/mrm.20508.

Jespersen, S.N., Leigland, L.A., Cornea, A., Kroenke, C.D., 2012. Determination of axonal and dendritic orientation distributions within the developing cerebral cortex by diffusion tensor imaging. IEEE Transactions on Medical Imaging 31, 16–32. doi:10.1109/TMI.2011.2162099.

Jones, D.K., 2014. Gaussian modeling of the diffusion signal, in: Johansen-Berg, H., Behrens, T.E.J. (Eds.), Diffusion MRI. second ed.. Academic Press, London. chapter 5, pp. 87–104. doi:10.1016/B978-0-12-396460-1.00005-6.

Kaden, E., Kelm, N.D., Carson, R.P., Does, M.D., Alexander, D.C., 2016. Multi-compartment microscopic diffusion imaging. NeuroImage 139, 346–359. doi:10.1016/j.neuroimage.2016.06.002.

Lätt, J., Nilsson, M., Wirestam, R., Ståhlberg, F., Karlsson, N., Johansson, M., Sundgren, P.C., van Westen, D., 2013. Regional values of diffusional kurtosis estimates in the healthy brain. Journal of Magnetic Resonance Imaging 37, 610–618. doi:10.1002/jmri.23857.

Mohammadi, S., Tabelow, K., Ruthotto, L., Feiweier, T., Polzehl, J., Weiskopf, N., 2015. High-resolution diffusion kurtosis imaging at 3T enabled by advanced post-processing. Frontiers in Neuroscience 8. doi:10.3389/fnins.2014.00427.

Veraart, J., Poot, D.H.J., Van Hecke, W., Blockx, I., Van der Linden, A., Verhoye, M., Sijbers, J., 2011. More accurate estimation of diffusion tensor parameters using diffusion kurtosis imaging. Magnetic Resonance in Medicine 65, 138–145. doi:10.1002/mrm.22603.

Zhang, H., Hubbard, P.L., Parker, G.J.M., Alexander, D.C., 2011. Axon diameter mapping in the presence of orientation dispersion with diffusion MRI. NeuroImage 56, 1301–1315. doi:10.1016/j.neuroimage.2011.01.084.

Zhang, H., Schneider, T., Wheeler-Kingshott, C.A., Alexander, D.C., 2012. NODDI: Practical in vivo neurite orientation dispersion and density imaging of the human brain. NeuroImage 61, 1000–1016. doi:10.1016/j.neuroimage.2012.03.072.
